# Multi-sensor geolocators unveil global and local movements in an Alpine-breeding long-distance migrant

**DOI:** 10.1186/s40462-023-00381-6

**Published:** 2023-04-05

**Authors:** Yann Rime, Raphaël Nussbaumer, Martins Briedis, Martha Maria Sander, Dan Chamberlain, Valentin Amrhein, Barbara Helm, Felix Liechti, Christoph M. Meier

**Affiliations:** 1 Department of Bird Migration, Swiss Ornithological Institute, Seerose 1, Sempach, CH-6204 Switzerland; 2 Department of Environmental Sciences, Zoology, University of Basel, Basel, CH-4051 Switzerland; 3 Cornell Lab of Ornithology, Ithaca, NY 14850 USA; 4 Institute of Biology, University of Latvia, Riga, LV-1004 Latvia; 5 Department of Life Sciences and Systems Biology, University of Turin, Via Accademia Albertina 13, Turin, IT-10123 Italy

**Keywords:** Multi-sensor loggers, Geolocators, Atmospheric pressure, Migratory behaviour, Local movements, Trans-saharan migrant

## Abstract

**Background:**

To understand the ecology of long-distance migrant bird species, it is necessary to study their full annual cycle, including migratory routes and stopovers. This is especially important for species in high-elevation habitats that are particularly vulnerable to environmental change. Here, we investigated both local and global movements during all parts of the annual cycle in a small trans-Saharan migratory bird breeding at high elevation.

**Methods:**

Recently, multi-sensor geolocators have opened new research opportunities in small-sized migratory organisms. We tagged Northern Wheatears *Oenanthe oenanthe* from the central-European Alpine population with loggers recording atmospheric pressure and light intensity. We modelled migration routes and identified stopover and non-breeding sites by correlating the atmospheric pressure measured on the birds with global atmospheric pressure data. Furthermore, we compared barrier-crossing flights with other migratory flights and studied the movement behaviour throughout the annual cycle.

**Results:**

All eight tracked individuals crossed the Mediterranean Sea, using islands for short stops, and made longer stopovers in the Atlas highlands. Single non-breeding sites were used during the entire boreal winter and were all located in the same region of the Sahel. Spring migration was recorded for four individuals with similar or slightly different routes compared to autumn. Migratory flights were typically nocturnal and characterized by fluctuating altitudes, frequently reaching 2000 to 4000 m a.s.l, with a maximum of up to 5150 m. Barrier-crossing flights, i.e., over the sea and the Sahara, were longer, higher, and faster compared to flights above favourable stopover habitat. In addition, we detected two types of altitudinal movements at the breeding site. Unexpected regular diel uphill movements were undertaken from the breeding territories towards nearby roosting sites at cliffs, while regional scale movements took place in response to local meteorological conditions during the pre-breeding period.

**Conclusion:**

Our data inform on both local and global scale movements, providing new insights into migratory behaviour and local movements in small songbirds. This calls for a wider use of multi-sensor loggers in songbird migration research, especially for investigating both local and global movements in the same individuals.

## Background

Understanding movements and identifying positions of migratory animals throughout their annual cycle is a prerequisite to assess the spatial and temporal aspects relevant to species conservation [1–3]. Annual movements allow migratory species to optimize the use of spatially and temporally limited resources [4]. However, migration across biomes involves vulnerability to various environmental changes during the annual cycle, especially in the case of long-distance migratory birds [5, 6]. Environmental conditions at the non-breeding site, such as drought and land-use changes in the Sahel region, impact populations of palearctic-breeding species [7–9]. Nevertheless, habitat and climate changes at the breeding site are also prime drivers of population trends in long-distance migrants [10–13]. While connecting breeding and non-breeding sites at a population level allows the assessment of sensitivity to site-specific changes, migration represents a critical phase. It is thus essential to describe individual migratory flights and stopover behaviour to fully understand the entire annual cycle of migratory species.

Landbirds migrating between Europe and Sub-Saharan Africa face major spatial and temporal challenges. First, they must cross the Mediterranean and the Sahara Desert, which are major ecological barriers between their breeding and non-breeding sites [14–16]. Barrier-crossing strategies vary within and between species [17–19]. Non-stop flights between breeding and non-breeding sites occur in some trans-Saharan migrants [20], even in small passerines [21]. While some large soaring birds tend to prioritize longer diurnal flights and avoid crossing broad surfaces of sea [22], direct or partial sea crossings are common in many wing-flapping species [23, 24], especially in songbirds [25, 26]. In this case, Mediterranean islands serve as important stopover sites for birds crossing large waterbodies [27] and many species land during the day when crossing the Sahara [28]. However, even small songbirds can cross the desert in a single non-stop flight [29]. Prolonged nocturnal migratory flights into daytime are common [30–32] and lead to behavioural adjustments, such as increased flight altitude during the day [33]. Similarly, flight altitudes tend to be higher over the Sahara Desert than during the rest of migration [34]. Nevertheless, due to technical limitations, vertical flight behaviour remains poorly understood in small songbirds.

Studying the full lifecycle of alpine species is crucial to understand their responses to environmental change, especially given the recent marked climate and habitat changes at high elevations [35]. In birds inhabiting mountain habitats, altitudinal movements occur not only during migration, but also at stationary sites. Typically, species breeding in highly seasonal environments, such as alpine habitats, must adjust their migratory and breeding timing to snowmelt and optimal availability of food resources [36–38]. In this regard, seasonal local altitudinal movements allow shifting resources across an elevational gradient to be tracked [39]. Diel altitudinal movements may also be aimed at tracking food resources or at coping with adverse meteorological conditions [40].

In the Northern Wheatear *Oenanthe oenanthe*, all populations migrate to sub-Saharan Africa, including those breeding in the Alps, but also those in Greenland or Alaska [41–43]. Populations from continental Europe have a shorter journey to Africa with longer stopovers [44, 45]. Light-level geolocation previously allowed the identification of non-breeding regions of Central European populations in the western Sahel [45, 46]. The Mediterranean has been identified as an important stopover area [26, 45]. However, due to a north-south migration around the time of equinox, when light recordings are not latitudinally informative, light-level geolocation has often resulted in poor estimates of the stopover locations, which calls for new methods to describe migration patterns (e.g., [47]).

In the Alps, Northern Wheatears face variable snowmelt and weather conditions upon arrival and tend to breed later than other central European populations [48, 49]. A potential mis-adaptation to a changing phenology of spring green-up has been suggested [45, 49], and the species is currently undergoing a broad-scale upward elevational shift [50, 51]. The phase between arrival and breeding is critical in high elevation birds [52, 53] and remains poorly understood. This advocates the investigation of movements not only during migration, but also at the breeding site.

Here, we studied migratory and local movements of Northern Wheatears from the Alps throughout the annual cycle using multi-sensor loggers recording light intensity and atmospheric pressure. Such devices have recently opened up new opportunities for research on the migration of small-bodied birds [32, 54, 55]. While informing on the altitude and duration of the stationary phases and of migratory or local flights [32, 54, 55], atmospheric pressure data also allow the geographic position of birds to be located during their stationary periods with a higher precision than light-level geolocation [56, 57]. We used atmospheric pressure to describe migration routes, stopover, and non-breeding locations as well as individual migration timing with an unprecedented precision in a small songbird. Furthermore, we analysed flight behaviour in relation to barrier-crossing migratory flights. We also detected two types of local movements at the breeding site: pre-breeding local altitudinal movements were undertaken in response to meteorological conditions and, more surprisingly in this territorial species [58–60], unexpected small-scale altitudinal movements occurred at the breeding site. In this study, we showcase that atmospheric pressure data can simultaneously unravel both global- and local-scale movements in small songbirds.

## Methods

### Geolocators and deployment

We fitted multi-sensor geolocators (GDL3-PAM, Swiss ornithological institute, 1.2 g with harness) on Northern Wheatears at two study areas: one in the Swiss Alps (Val Piora, 46°33’N, 8°42’E, 1850 to 2200 m a.s.l.) and one in the Italian Alps (Val Troncea, 44°57′N, 6°56′E, 1900 to 2700 m a.s.l.). These devices mounted using a leg-loop harness[61] record ambient light intensity, atmospheric pressure, and temperature. In this species, both males and females usually return to the same breeding territories, allowing recapture in subsequent years [58, 62–64]. All birds were individually marked with a colour combination of three plastic rings and a metal ring. Birds were trapped with a trap placed at the entrance of the nest cavity while feeding the chicks (both for the first capture and recapture), or with baited spring traps in the territory (recapture only). Between 2016 and 2020, we ringed 301 adult birds in June and July at the Swiss site. We equipped 54 individuals with GDL3-PAM geolocators (2016, 2018, 2019 and 2020). 140 birds were ringed but not tagged and served as a control group. 47% of the control birds (66/140) and 37% of the GDL3 PAM (20/54) were observed in the year after tagging. In addition, we equipped 40 adults in 2019 and 2020 in the Italian Alps, with a control group of 23 birds; here, 48% of the control birds (11/23) and 22.5% of the GDL3 PAM (9/40) were observed in the year after tagging. The mean ± standard deviation body mass of adults at the Swiss site was 24.6 ± 1.6 g (range 20.0–30.0 g) and 23.6 ± 1.9 g at the Italian site (range 19.8–32.0 g). The device always amounted to less than 5% of the body mass of the tagged birds [65]. We compared return rates of the tagged and control individuals, under the same resighting effort and handling procedure except for fitting the loggers, using a Fisher’s exact test (p = 0.47 for the Swiss site, p = 0.19 for the Italian site) and a 2-sample test for equality of proportions with continuity correction (X^2^ = 1.23, p = 0.27 and X^2^ = 3.23, p = 0.072, respectively). A high proportion of these experimental devices failed a few weeks after tagging because of battery issues (n = 16/24, mostly those fitted in 2016, 2018 and 2019), resulting in recording of only partial tracks. Here, we considered only the tracks with at least a full autumn migration (n = 8 for autumn migration, n = 6 for non-breeding site data, n = 4 for spring migration; Table 1).

**Table 1:** Summary of the general information, migration schedule and flight performance of Northern Wheatears tagged with multi-sensor geolocators showing full annual cycle (n = 4) and fall migration (n = 8)

ID	26IM	26IL	26HS	20TJ	16IQ	24IS	24TJ	24EA
Sex	M	M	F	F	F	M	M	F
First data	14.07.20	14.07.20	07.07.20	01.08.18	01.07.17	01.08.19	31.07.20	20.07.19
Breeding site	CH	CH	CH	CH	CH	CH	IT	IT
Last data	28.05.21	30.06.21	23.06.21	08.11.18	28.01.17	16.10.20	05.06.20	31.10.19
**Autumn migration**								
Departure breeding site	16.09.20	10.09.20	16.09.20	10.09.20	12.09.20	15.09.20	12.09.20	15.09.20
Duration (days)	23	26	48	32	25	-	37	-
Duration of stopover in N Africa	9	11	24	21	12	10	16	25
Arrival non-breeding site	09.10.20	06.10.20	03.11.20	12.10.18	07.10.16	-	19.10.20	-
Mediterranean stopover site	Spanish coast	Sardinia	Corsica, Sardinia	None	None	None	Sardinia	Baleares
Number of migratory flights	15	13	17	11	20	12	17	12
Total migration distance	4625	4055	4716	4032	4241	4296	4121	3733
Cumulative flight hours	87	69.5	103.5	79	90	85.5	73	79.5
Mean ground speed per flight	45.3	54.5	39.7	47.2	39.4	42.8	48.4	46.2
Mean flight altitude per flight	1287	1478	1270	1559	1276	1554	1229	1287
Max flight altitude	4120	4530	4157	4764	4250	4475	4600	3662
**Spring migration**								
Departure from the NB site	01.04.21	04.04.21	04.04.21	-	-	-	05.04.21	-
Duration (days)	32	22	39	-	-	-	29	-
Duration of stopover in N Africa	18	13	13	-	-	-	16	-
Arrival at the B site	03.05.21	26.04.21	13.05.21	-	-	-	04.05.21	-
Mediterranean stopover site	None	Baleares	Sardinia	-	-	-	None	-
Number of migratory flights	11	15	14	-	-	-	13	-
Total migration distance	4581	4873	3736	-	-	-	4415	-
Cumulative flight hours	78.5	104.5	74	-	-	-	78	-
Mean ground speed per flight	46.4	42.8	42.4	-	-	-	52	-
Mean flight altitude per flight	1387	1667	1631	-	-	-	1454	-
Max flight altitude	4567	5148	3725	-	-	-	4306	-

### Trajectory reconstruction

We modelled the trajectory of each track following the approach presented in Nussbaumer et al. (2023, [66]) and using the R package GeoPressureR (version 2.7, [67]. All analyses were performed in R version 4.2.0 [68]. We briefly describe the main steps of the approach below.

First, we identified stationary periods, when a bird was presumed to remain at the same location at the resolution of our model (0.25°, i.e. 27 km). We manually labelled the geolocator pressure measurements: stationary periods were characterized by a limited variation in consecutive pressure measurements indicating an absence of change in altitude, while migratory flights typically displayed a clear drop in atmospheric pressure, corresponding to altitude gain in-flight.

Second, we constructed separate probability maps based on atmospheric pressure and light intensity data (sunrise and sunset times) for position estimates of each bird during each stationary period. For the pressure-based maps, the time series of the geolocator pressure measurements during stationary periods were matched with the one-hour ERA5 surface level reanalysis dataset (spatial resolution: 0.25 × 0.25°) to produce a likelihood map of the geolocator’s position [57]. The likelihood map produced included the information of both the temporal variation of pressure and the absolute values of pressure corresponding to the altitudinal range within each grid cell.

For the light-based maps, we calculated likelihood estimates following Nussbaumer et al. (2022a, [57]). We used an “in-habitat” calibration from the equipment and retrieval periods [69–71], fitting the distribution of zenith angle with a kernel density estimation. The likelihood maps of twilights belonging to the same stationary period were aggregated with a log-linear pooling.

Finally, we constructed the trajectories of each bird following the Hidden Markov Model presented in Nussbaumer et al. (2023, [66]). The observation model consisted of the likelihood maps generated from pressure and light data. The movement model used the information of flight duration derived from the labelling together with wind data, so that the parametric equation of movement was defined on airspeed. This parametric equation was defined as the cubic root of the mechanical power required for the average airspeed computed for a transition, accounting for a typical Northern Wheatear size and shape. A low airspeed threshold of 20 km/h was used to account for potential short local or exploratory flights. Using this model, we generated (1) the marginal probability map of the position of each stationary period, (2) the most likely migration trajectory of the birds, and (3) 100 random simulations of the trajectories. We refer readers to Nussbaumer et al. (2023, [66]) for further details on the implementation of this method.

Data, code and parameter values used in this study are available under the DOI: 10.5281 at https://zenodo.org/record/7471405 [72].

### Description of flight behaviour

For each migratory flight, we extracted (1) flight duration, (2) maximum and mean flight altitude, as well as (3) positive altitudinal change during the flight using a standard barometric equation while correcting for the temporal variation of pressure from the ERA 5 data at the most likely location. We then calculated (4) ground speed, wind support and flight distance for each of the 100 random simulations. We then classified the flights in five categories (above continental Europe, the Mediterranean Sea, the Atlas region, the Sahara Desert, and the Sahel region) and calculated the mean and standard deviation of the different variables for each of these flight categories. We also distinguished between flights during autumn and spring migration.

## Results

### Migration patterns: timing and locations

**Fig. 1:**
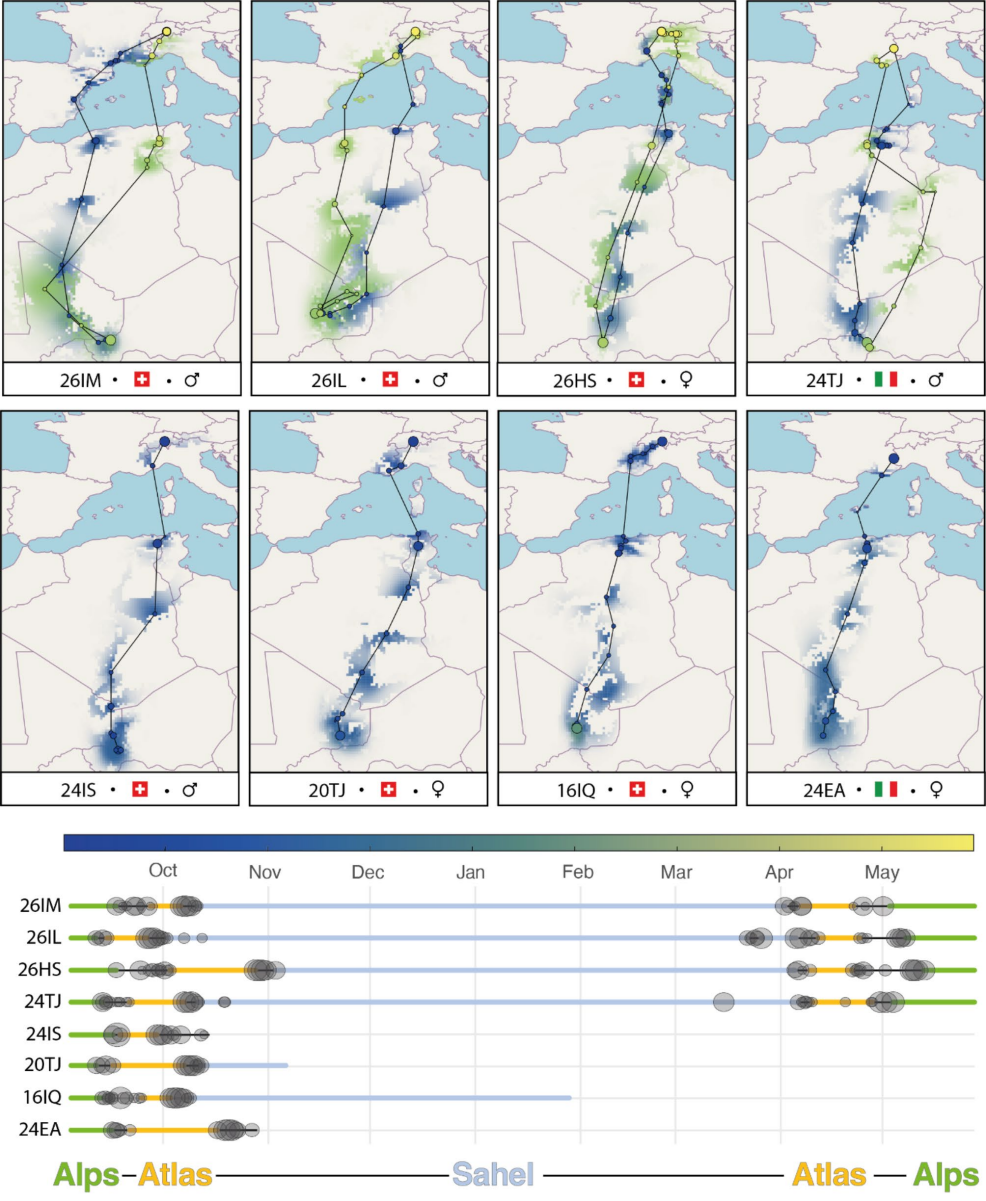
a. Most likely trajectories and stationary locations of the Northern Wheatears equipped with multi-sensor geolocators in the Swiss Alps (26IM, 26IL, 26HS, 24IS, 20TJ, 16IQ) and Italian Alps (24TJ, 24EA). For visual purposes, the colour scale for all stationary sites was normalized to a maximal value of 1 and represents the marginal probability of the position of the bird. Autumn migration appears in blue and spring migration in green b. Time series of the individuals with breeding site (green), migration (yellow) and non-breeding sites (blue). The grey circles show migratory flights. The size of the circles is proportional to the flight duration

**Fig. 2:**
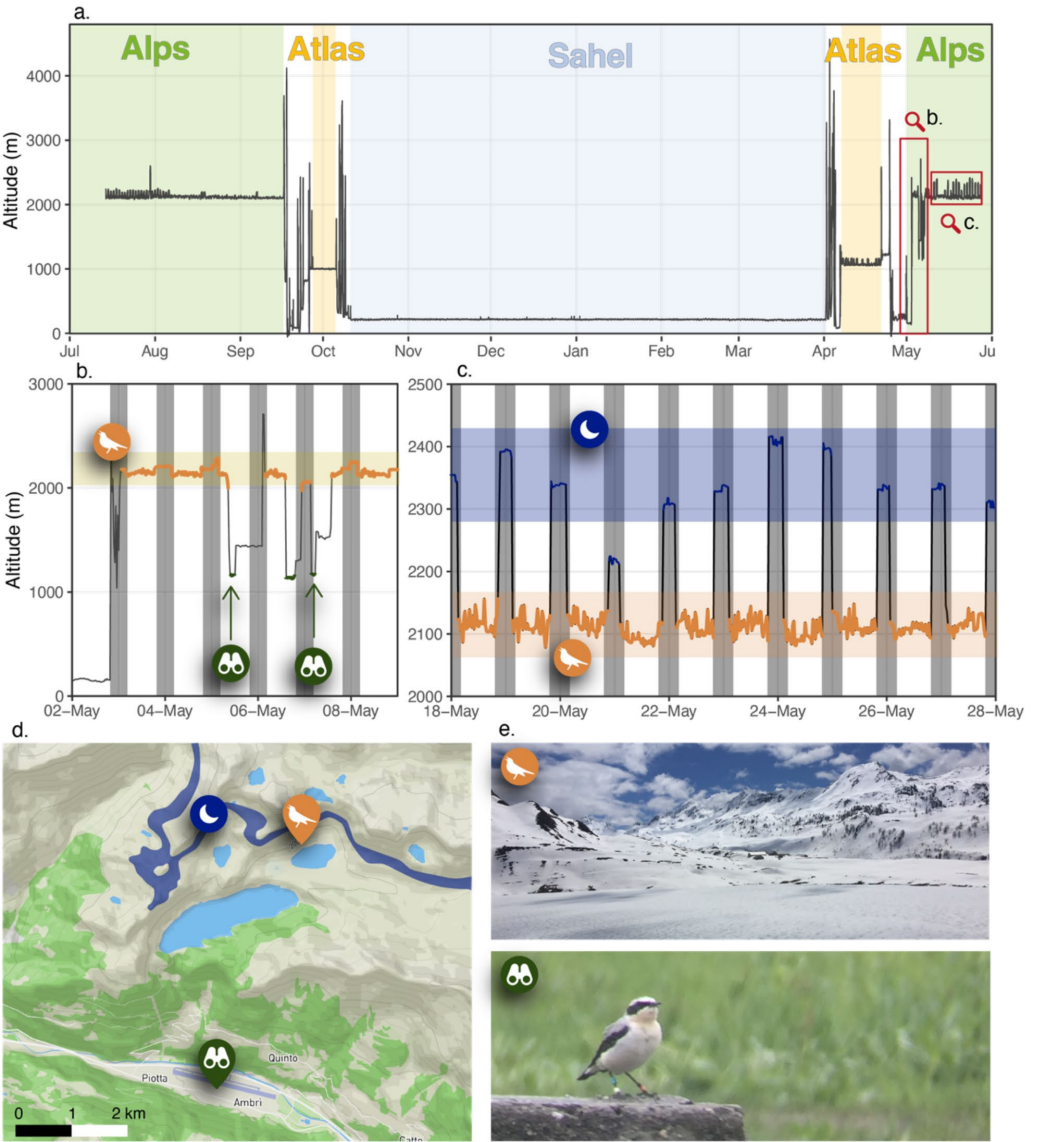
a. Altitudinal profile of the male 26IM during the annual cycle. The green transparent area shows the breeding site, yellow the stopover in the Atlas, and blue the non-breeding site. The red box b. refers to the zoom on Fig. 2b and the red box c. to the zoom on Fig. 2c b. Altitudinal profile of the bird 26IM between 2 May and 9 May 2021 (red box b on Fig. 2a). The orange line is the measured position of the bird when it was at the breeding territory. The dark-green line shows the residency at lower elevation in the nearby valley, highlighted with the binocular symbol for the two occasions when the bird was observed directly c. Altitudinal profile of the bird 26IM between 18 May and 28 May 2021 (red box c on Fig. 2a). The orange shade shows the area of the breeding territory and the orange line the measured position of the bird within the territory. The blue lines show the measured position of the bird at night. The shaded blue area with the moon symbol shows the area where the bird roosts, corresponding to the blue area on Fig. 2d d. Map of the study region showing the breeding territory (orange), the roosting elevational area near the breeding territory (blue) and the area where the bird was observed in the nearby valley during bad weather events (Fig. 2b) e. Breeding territory at the time of arrival and photography of the male 26IM in the nearby valley on 5 May 2021

**Fig. 3:**
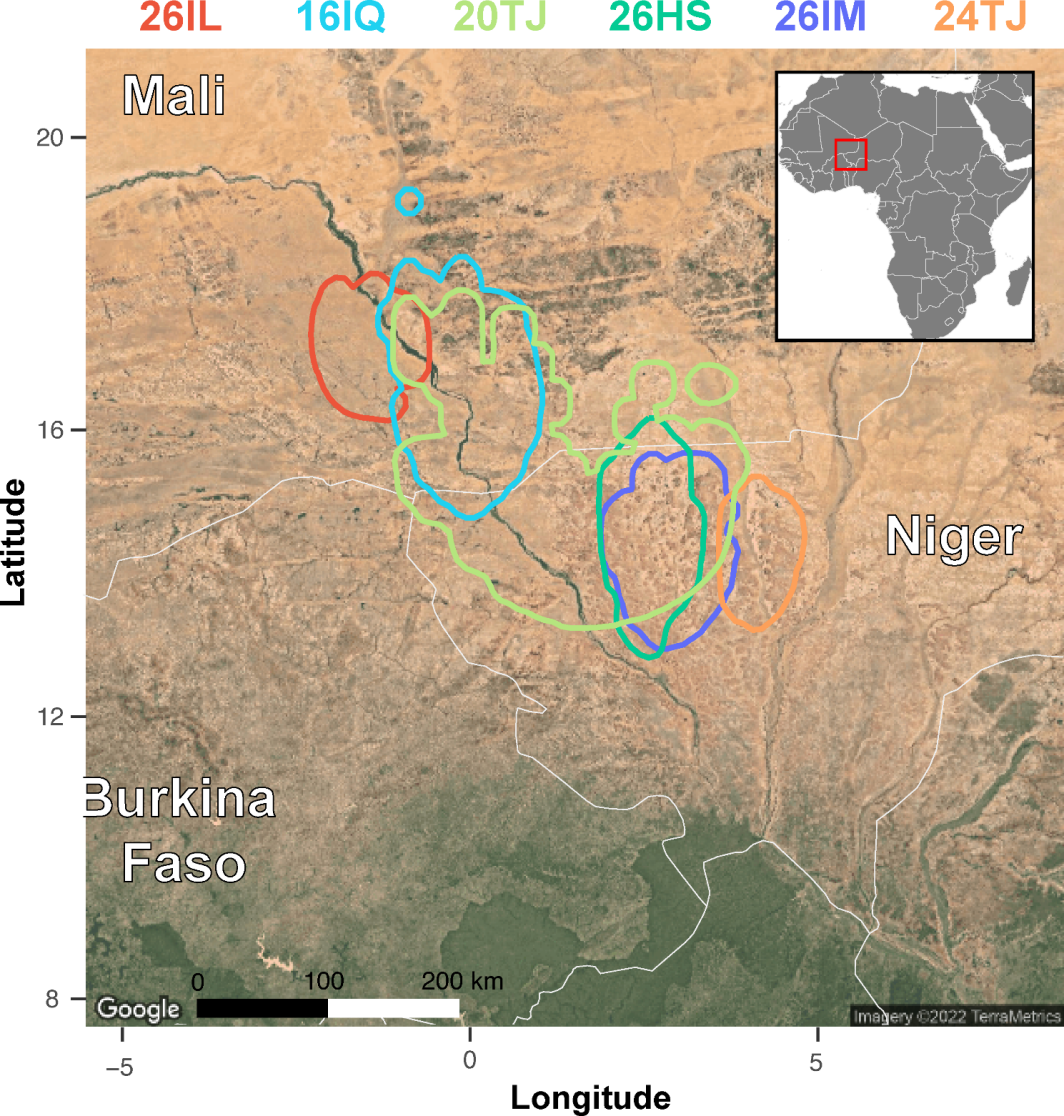
Most likely locations of the non-breeding sites of six individuals between E Mali, W Niger and N Burkina Faso in Western Africa. The delineated areas correspond to the 99th quantile of the marginal probability distribution of stationary locations extracted from the hidden Markov model. Because the device was operational for a shorter duration, the estimated area for the individual 20TJ is larger

Tracks and migration timing are summarized in Fig. 1, and general information is given in Table 1. An example of a full annual altitudinal profile based on atmospheric pressure data is given in Fig. 2a. The tagged individuals stayed at the breeding site in the post-breeding period until departure to migration. In all years, they departed from the breeding sites between the 10th and the 16th of September (n = 8, Fig. 1). They spent 0 to 9 days (mean ± sd = 3.5 ± 3.2) between the breeding site in the Alps and the Mediterranean coast (Liguria, Italy) before starting to cross the Mediterranean. In 2020, a female from Switzerland (26HS, Fig. 1) spent several days at high elevations in the western Alps (3040 to 3200 m) before flying over the Mediterranean to Corsica directly from this alpine site. One bird from Italy (24TJ, Fig. 1) crossed the Mediterranean directly from the breeding site and another one stopped on the coast over the day. Four birds landed briefly in Sardinia, Corsica or the Balearic Islands in the Mediterranean in autumn, and four birds crossed the Mediterranean without stopping over. A female briefly landed on both Corsica and Sardinia (26HS, Fig. 1). Overall, birds crossed the Mediterranean quickly (mean ± sd = 2 ± 2.4 days, median = 1 day), but then spent a long stopover of 9 to 25 days (mean ± sd = 16 ± 6.5) in the Atlas highlands of northern Africa before crossing the Sahara in the second phase of autumn migration. The crossing of the Sahara was direct in autumn, consisting of 4 to 6 typically long nocturnal flights, with stops during the day. The birds thereafter spent a few days in the Sahel, interspersed with shorter flights (further details about flight durations are given under the “flight performance” result section), before settling at a single site for the entire non-breeding period. The four individuals that provided data for a full cycle were stationary for 165.3 ± 9.4 days on sites located close to each other between eastern Mali and western Niger (Figs. 1 and 3).

Spring migration (n = 4, year 2021) started between April 1st and 5th. However, one male (26IL, Fig. 1) initiated two earlier migratory-like flights to the North-East (duration of 8 h with 27 km/h perpendicular wind from the North-West and 10 h with 48 km/h supporting wind from the South-West), but then returned to the non-breeding area (duration of 6 h with 25 km/h perpendicular wind from the North-East and 8 h with 35 km/h supporting wind from the North-East), before departing finally on April 4th. The four birds crossed the Sahara in a similar way in spring compared to autumn, in 3 to 5 nights with daytime stopovers. A main stopover again took place in the highlands of Northern Algeria in spring, with a duration of 15 ± 2.4 days. Mediterranean Islands were used for short stops in two cases (Table 1; Fig. 1).

### Migratory flight performance

**Fig. 4:**
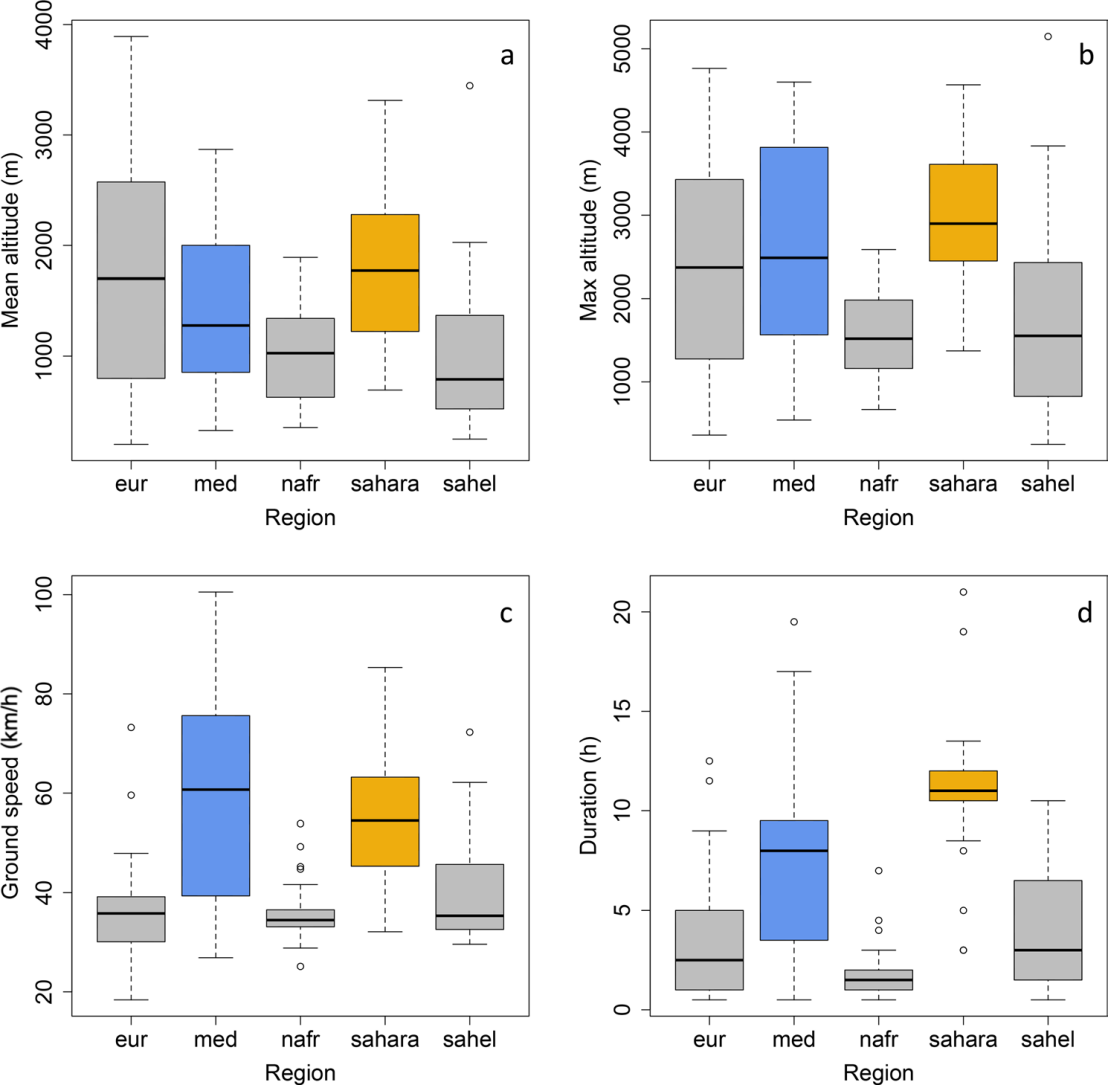
Mean flight altitude per flight (a), maximum flight altitude per flight (b), ground speed (c) and flight duration (d) over continental Europe (eur), the Mediterranean Sea (med), the Atlas region in Northern Africa (nafr), the Sahara Desert (sahara), and the Sahel region (sahel)

During autumn (n = 8 individuals) and spring migration (n = 4 individuals), we described 167 migratory flights (117 in autumn and 50 in spring) that were initiated at dusk and were only rarely prolonged into the following day (n = 11 flights longer than 12 h and 4 flights longer than 14 h, maximum = 21 h). The flights had the following characteristics (mean ± sd): duration 5.9 ± 4.6 h, maximum altitude per flight 2294 ± 1172 m and up to 5150 m, average altitude per flight 1409 ± 796 m, positive altitudinal change 2298 ± 1902 m, positive altitudinal change per flight hour 405 ± 239 m, ground speed 45 ± 16 km/h, wind support 7 ± 10 km/h and distance 302 ± 296 km. Flight duration (Fig. 4c) was longer over the Mediterranean and Sahara (n = 45) than over continental Europe (n = 40), the Atlas (n = 27) and the Sahel (n = 34). Maximum and mean flight altitude (Fig. 4a, b) were higher above the Sahara and Europe than above the Atlas and the Sahel, but more variable above the Mediterranean. Ground speed (Fig. 4d) was faster above the Mediterranean and Sahara than above Europe, the Atlas and the Sahel. Positive altitudinal change was higher above the Mediterranean (3116 ± 836 m) and the Sahara (3992 ± 1659 m) than above Europe (1303 ± 1233 m), the Atlas (836 ± 607 m) and the Sahel (1811 ± 1578 m), but positive altitudinal change rate per hour did not show any particular pattern between long barrier-crossing flights and other flights (Europe 352 ± 254 m/h, Mediterranean 415 ± 178 m/h, Atlas 469 ± 345 m/h, Sahara 369 ± 126 m/h, Sahel 456 ± 259 m/h). Wind support was generally used above the Mediterranean (10 ± 10 km/h) and the Sahara (12 ± 11 km/h), but less above Europe (1 ± 4 km/h), the Atlas (6 ± 9 km/h) and the Sahel (7 ± 9 km/h). Flight distances were longer over the Mediterranean (481 ± 317 km) and over the Sahara (609 ± 261 km) and much shorter in Europe (122 ± 109 km), the Atlas (69 ± 56 km) and the Sahel (169 ± 136 km).

### Local altitudinal movements at the breeding site

Individuals arrived at the breeding territory between April 26th and May 13th (n = 4). The behaviour upon arrival in the breeding region differed between individuals. One Swiss male (26IM) moved on three occasions from the breeding site to the adjacent valley, 5 km further and 1000 m lower than the breeding territory. This coincided with an adverse weather event with snowfall at the breeding site (Fig. 2b, d and e). This behaviour was confirmed twice with visual observations in the field. The bird came back to the territory between these foraging excursions, with one flight measured at 2600 m a.s.l. The third male also performed altitudinal movements in the nearby Alps. The latest arrival was recorded for the female, which stayed at high elevation sites (1640 to 2085 m) in the Alps between April 25th and May 8th before reaching the breeding site on May 13th. The male from Italy (24TJ) did not undertake local altitudinal movements to the valley upon arrival (Fig. 1).

During their presence at the breeding site, all four birds undertook diel altitudinal movements from the breeding and foraging territories towards roosting sites, located between 50 and 300 m higher (example for the male 26IM, Fig. 2c). In the female 26HS, this behaviour occurred in the post-breeding season 2020, as well as in the pre-breeding season 2021, but was interrupted when she started incubating in June. The tag was retrieved when the female was feeding the chicks. The males continued to commute at night while breeding.

## Discussion

We used atmospheric pressure measurements, combined with light-level geolocation, to describe local and global movements of Alpine Northern Wheatears, one of the few long-distance migrants breeding in high-alpine habitats, throughout the annual cycle. We located the main stopover area in the northern African highlands, underlining the importance of this region in autumn and spring. We refined the knowledge of the location of non-breeding sites in the Alpine population, within a restricted area in the Western Sahel and detected residency at a single site during winter. While migration timing showed synchrony between the tagged birds, we highlighted faster and longer flights over migratory barriers, especially the Sahara, compared to areas with favourable stopover opportunities. Flight altitude was typically higher above the desert. Moreover, we described local altitudinal movements at the breeding site, with occasional movements to an adjacent valley in response to weather conditions in the pre-breeding season, as well as unexpected regular diel commuting behaviour at the nesting site.

The time window for the initiation of autumn migration across different years was surprisingly narrow compared to short-distance high-elevation migratory species with higher variability in departure date [39, 73]. Compared to previous results from light-level geolocation suggesting a main stopover of the Alpine population in the central and western Mediterranean [26, 45, 46], the identification of a major stopover region in the Atlas highlands of Northern Africa underlines the advantages of using atmospheric pressure to estimate stationary locations [57]. The open agricultural and pastoral land uses typical of these regions obviously serve as refuelling habitats between barrier crossings. This complements the findings of Maggini and Bairlein (2011, [74]), who described Northern Wheatears at a lowland spring stopover on the edge of the Sahara as not having sufficient body condition to subsequently cross the Mediterranean without refuelling. These birds however did not remain at this site, but most likely continued towards further stopover sites, probably in the Atlas highlands further north.

All four individuals tracked for an entire annual cycle stayed at a single location throughout the boreal winter, without any altitudinal movement detected in the pressure time series within the non-breeding region. However, previous light-level geolocation studies identified movements in some individuals within the non-breeding region [26, 45]. Other Afro-Palearctic migrants, especially species linked to wetlands such as the Great Reed Warbler *Acrocephalus arundinaceus*, shift between successive non-breeding sites to track resources [75, 76]. The residency at a single non-breeding site in the Wheatears we tracked is likely related to the site-fidelity and territorial behaviour of the species on the wintering grounds [77].

Migration of the Central European populations of the Northern Wheatear is north-south oriented, resulting in flights above the Mediterranean as the shortest route [26, 44, 45]. The species can fly non-stop for more than 2500 km over the Atlantic Ocean [43, 78], and Cyprus Wheatears *Oenanthe cypriaca* can reach their non-breeding sites in a non-stop flight [21]. Hence, one could expect non-stop Mediterranean crossing in Northern Wheatears. However, in autumn and spring some birds landed on the Mediterranean islands, or flew further west towards the Iberian Peninsula, where the sea is narrower. This underlines the role of small islands for migratory songbirds in the central Mediterranean [27], although it remains unclear whether these short stopovers were intentional (the bird knew about the land mass and directed its flight towards it) or happened opportunistically (the bird saw the land mass and decided to stop).

Northern Wheatears adapted their migratory flights in relation to barrier crossing. Some migratory songbirds, such as Tawny Pipits *Anthus campestris* and Great Reed Warblers, regularly prolong their nocturnal migratory flights into the following day [32, 33]. Nevertheless, the tracked Wheatears mostly performed nocturnal migratory flights interspersed with stopovers during the day. These flights were, however, notably longer and faster while crossing barriers such as the Mediterranean and the Sahara, than were the flights performed closer to the breeding and non-breeding sites and above the favourable Atlas region in Northern Africa. Barrier-crossing flights were generally wind-supported. They showed altitudinal fluctuations and hence a higher total positive altitudinal change than shorter migratory flights, but not a higher climb rate per hour. Müller et al. (2018), Schmaljohann et al. (2011) and Schmaljohann & Naef-Daenzer (2011) [79–81] described departures earlier in the evening in the case of barrier-crossings flights towards the Atlantic Ocean and Greenland. Here, the nature of the barrier differed: while crossing the Mediterranean and the Sahara, birds can stop over when needed, which is not possible over the ocean. Numerous short flights interspersed with daily stopovers took place before reaching the non-breeding site in the Sahel region at the end of autumn migration, as well as before returning to the breeding site in spring. Such short flights also occurred in the Atlas region of Northern Africa, as suggested by Maggini and Bairlein (2011, [74]). Some birds also stayed at different locations in the Alps before autumn migration and after spring migration. More surprisingly, one individual performed migratory flights and returned to the non-breeding area before undertaking the actual spring migration (this event was apparently linked with wind support conditions); using only light-level data in this case would cause imprecision of several days when inferring migration timing from changes in light-level stationary locations [26]. Birds generally flew at higher elevations above the Sahara than during the rest of the migration and remained stationary in the heat of the day. As an open-ground species, the Northern Wheatear is more likely to stop over in the desert than are wetland species [30] or forest species [29]. High elevation flights were recorded at night and took advantage of supportive winds, thereby suggesting that heat avoidance during daylight hours would itself not be sufficient to explain high-altitude flights over the desert in songbirds [33].

Our data also unveiled local altitudinal movements in Northern Wheatears. Such movements typically occurred in two distinct phases in the pre-breeding and breeding seasons. First, occasional local altitudinal movements of larger amplitude were undertaken in the pre-breeding period. Similar early-season altitudinal movements occur in other alpine migratory species such as the Ring Ouzel *Turdus torquatus alpestris* [39]. However, in the case of the Northern Wheatear, the movements were irregular and appeared to be an emergency response to extreme meteorological events such as late and intense snowfalls upon arrival at the breeding site. One bird flew at 2600 m between the foraging site in the valley and the breeding territory, indicating that the individual did not minimize the elevational difference while commuting between alternative foraging sites and the breeding territory.

A second type of altitudinal movement, unexpected in this territorial and breeding-site-faithful species [48, 59, 82], occurred at night, while birds flew up to roost, most likely in nearby cliffs, sometimes changing location after one to three nights at the same elevation. This behaviour was halted by the female during breeding but continued throughout the breeding season in males. It is unclear whether commuting aims to optimise temperature at night or to avoid predation, or a combination of both – such behaviour is a typical strategy to cope with cold nights in high-alpine passerines such as the White-Winged Snowfinch *Montifringilla nivalis* roosting in cliff crevices where the temperature remains higher than outside [40]. During the post-breeding season, Northern Wheatears remained at the breeding site until departure for migration, including the period of moult, as previously demonstrated for lowland populations [83]. This stresses the importance of suitable habitat throughout the period of presence at the breeding site.

## Conclusion

Our study provides novel insight into migration strategies, flight behaviour, barrier crossing and local altitudinal movements in a small songbird, including life history stages such as the transition between arrival and breeding, the post-breeding season, and moult. Movement behaviour was surprisingly variable in the Northern Wheatear, with almost no movement over more than five months at the non-breeding site, opposed to locally mobile behaviour at the breeding site and broader-scale movements during migration. This overview of the annual cycle calls for a wider use of pressure loggers to investigate the three-dimensional movements of songbirds at different spatial scales, especially using the method of positioning by correlating the atmospheric pressure measured on the bird with global atmospheric pressure data [57, 66].

## Data Availability

Data, codes, and parameter values used in this study are under the DOI: 10.5281 at https://zenodo.org/record/7471405 [72]
